# The Role of the HMGB1 C-Terminal Domain in Epithelial–Mesenchymal Transition and Invasion in 2D and 3D MDA-MB-231 Breast Cancer Models

**DOI:** 10.3390/ijms27073146

**Published:** 2026-03-30

**Authors:** Desislava Vladimirova, Shazie Yusein-Myashkova, Evdokia Pasheva, Iva Ugrinova, Jordana Todorova

**Affiliations:** Institute of Molecular Biology “Acad. Roumen Tsanev”, Bulgarian Academy of Sciences, 1113 Sofia, Bulgaria; desivladimirova@abv.bg (D.V.); shazi@abv.bg (S.Y.-M.); eva@bio21.bas.bg (E.P.)

**Keywords:** HMGB1, HMGB1ΔC, RAGE, epithelial-to-mesenchymal transition (EMT), 3D spheroid model, metformin

## Abstract

High-mobility group box 1 (HMGB1) is a multifunctional protein that operates both within the nucleus and as an extracellular signaling molecule. Its extracellular activity has been increasingly associated with cancer progression. Emerging evidence suggests that structural modifications of HMGB1, including C-terminal truncation, may alter its biological activity, though the underlying mechanisms remain largely unexplored. Here, we show that HMGB1, which lacks the entire C-terminal acidic tail, is associated with increased cellular plasticity and invasive potential through distinct signaling pathways not strictly dependent on RAGE (Receptor for Advanced Glycation End-product) under the tested conditions. Functional analyses indicate that this truncated form promotes epithelial–mesenchymal transition-related behaviors and activates downstream inflammatory signaling in a context-dependent manner. Notably, pharmacological intervention with metformin effectively suppressed responses to the full-length protein but was less effective against the tail-less variant, underscoring potential therapeutic challenges. These findings suggest an underappreciated regulatory role of the HMGB1 C-terminal domain in tumor aggressiveness.

## 1. Introduction

HMGB1 is a ubiquitously expressed, multifunctional nuclear protein with diverse roles in maintaining genomic architecture, regulating transcription, and orchestrating extracellular signaling under conditions of stress, inflammation, and cancer [1,2,3]. In its nuclear form, HMGB1 binds DNA and modulates chromatin structure, thereby influencing transcriptional responses. However, upon cellular stress, necrosis, or active secretion, HMGB1 is released into the extracellular space, where it acts as a potent damage-associated molecular pattern (DAMP) molecule [3]. Extracellular HMGB1 contributes to tumor progression through pleiotropic mechanisms, including stimulation of angiogenesis [4], immune cell recruitment [5], epithelial–mesenchymal transition (EMT), and metastasis [6,7].

Recent evidence suggests that HMGB1 undergoes post-translational modifications, including proteolytic truncation of its C-terminal acidic tail. This modification has been observed in inflammatory conditions such as arthritis, where HMGB1 with truncated C-terminal tail remains biologically active [8]. However, the functional consequences of this truncation remain controversial. Some studies report enhanced pro-inflammatory activity [9], while others suggest attenuated signaling [10]. In cancer, HMGB1 has been implicated in the induction of EMT, increased cell motility, invasion, and metastatic potential [11], yet the role of HMGB1ΔC in these processes has not been systematically investigated.

HMGB1 promotes EMT and invasion primarily via receptor for advanced glycation end-product (RAGE)-mediated activation of downstream signaling pathways, including NF-κB, which regulates the transcription of genes involved in motility and invasion [12]. Pharmacological interventions targeting HMGB1 signaling, such as metformin, have shown promise in reducing cancer aggressiveness, although the efficacy of these interventions in the context of HMGB1 truncation is unknown [13]. Here, we investigated the effects of removing the entire C-terminal acidic tail of HMGB1 (HMGB1ΔC) on epithelial–mesenchymal transition (EMT), cell motility, and invasion in MDA-MB-231 breast cancer cell line. Furthermore, we examined whether metformin could inhibit HMGB1ΔC-induced pro-carcinogenic phenotypes. Previous studies have shown that metformin can directly bind to the C-terminal acidic tail of HMGB1, but not to the A box or B box, thereby inhibiting HMGB1-induced inflammatory responses [14]. Our findings provide novel insights into the functional consequences of HMGB1 truncation in cancer progression, highlighting potential limitations of pharmacological interventions targeting HMGB1 signaling.

## 2. Results

### 2.1. HMGB1ΔC Induces Cell Motility Comparable to Full-Length HMGB1

**Figure 1:**
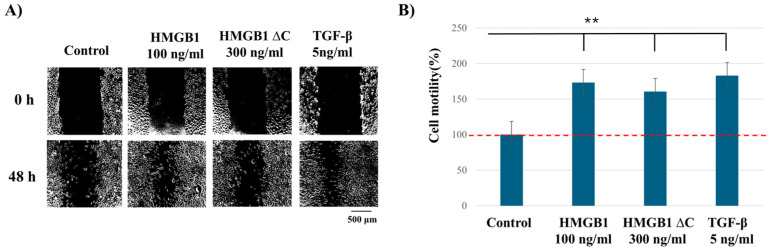
Effect of HMGB1, HMGB1ΔC, and TGF-β on MDA-MB-231 cell migration. (**A**) Cells were treated with the optimal concentrations of HMGB1, HMGB1ΔC, or TGF-β, and wound healing assays were performed. (**B**) Migration was quantified and normalized to the control, which was set as 100% wound closure. Data are presented as mean ± standard deviation (SD). Statistical analysis was performed using one-way ANOVA. Statistically significant differences are indicated with ** *p* < 0.01.

MDA-MB-231 cells were selected as the experimental model because they exhibit the highest basal expression of HMGB1, RAGE, and phosphorylated NF-κB p65, indicating constitutive activation of HMGB1-dependent signaling compared with the other breast cancer and non-tumorigenic cell lines. Using a model like this with constitutive HMGB1–RAGE–NF-κB activation offers advantages for studying exogenous HMGB1 by providing enhanced sensitivity and synergistic amplification. The MDA-MB-231 cell line was first tested for its migratory capacity using a scratch wound healing assay. To ensure that the observed gap closure was due to migration rather than cell proliferation, the assay was conducted under serum-starved conditions (0.1% FBS). Cells were treated with recombinant full-length HMGB1 (100–1000 ng/mL), HMGB1ΔC (100–1000 ng/mL), and transforming growth factor-β (TGF-β) as a positive control (1–10 ng/mL), and wound healing assays were performed. Optimal concentrations were determined as 100 ng/mL for HMGB1, 300 ng/mL for HMGB1ΔC, and 5 ng/mL for TGF-β (Figure S1A–C). All three treatments at the optimal concentrations—full-length HMGB1, HMGB1ΔC, and TGF-β—induced comparable increases in MDA-MB-231 cell motility, although significant differences were observed compared to control conditions, no statistically significant differences were detected between HMGB1, HMGB1ΔC, and TGF-β treatments (Figure 1). These data indicate that HMGB1ΔC is functionally capable of promoting cell migration similarly to full-length HMGB1 and the well-established EMT inducer TGF-β. To achieve equivalent migratory effects, a higher concentration of HMGB1ΔC (300 ng/mL) was necessary compared to full-length HMGB1 (100 ng/mL), suggesting that while the C-terminal tail enhances HMGB1’s potency, its absence does not abolish its pro-migratory activity.

Based on these results, all subsequent functional experiments were performed using these concentrations in MDA-MB-231 cells, providing a physiologically relevant model with active HMGB1-RAGE-NF-κB signaling.

### 2.2. HMGB1ΔC Enhances Mesenchymal Features and EMT-Associated Markers

**Figure 2:**
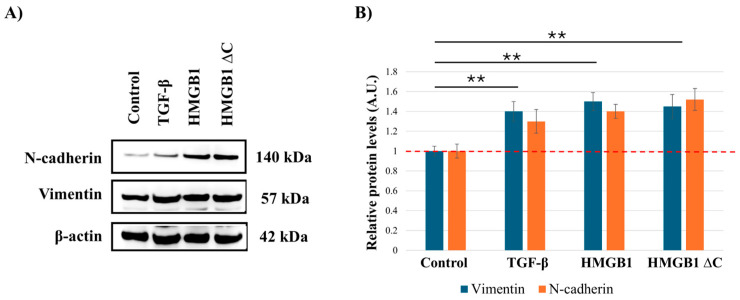
Analysis of EMT marker expression in MDA-MB-231 cells following treatment with HMGB1, HMGB1ΔC, and TGF-β. (**A**) Representative Western blot images showing changes in the expression levels of N-cadherin and vimentin in MDA-MB-231 cells treated with 100 ng/mL HMGB1, 300 ng/mL HMGB1ΔC, and 5 ng/mL TGF-β. (**B**) Quantification of protein expression based on densitometric analysis of Western blot signals from three independent experiments (n = 3) using ImageJ software. Data are presented as mean ± SD and expressed as fold change relative to the control (untreated) group, for which the expression level was set to 1. Statistical analysis was performed using one-way ANOVA. Statistically significant differences are indicated with ** *p* < 0.01. Values are presented in arbitrary units (A.U.).

To determine whether the observed increase in cell motility was associated with enhancement of EMT-associated traits, MDA-MB-231 cells were treated with the same concentrations from the wound healing assay, which were identified as the most optimal in inducing motility (100 ng/mL HMGB1, 300 ng/mL HMGB1ΔC, and 5 ng/mL TGF-β). Western blot analysis was performed to assess the expression of the mesenchymal markers N-cadherin and vimentin. The results demonstrated a marked upregulation of both proteins following treatment with HMGB1ΔC and HMGB1, as well as with recombinant TGF-β (Figure 2). These findings demonstrate that HMGB1ΔC, similarly to HMGB1 and the positive control TGF-β, can also further enhance mesenchymal characteristics.

### 2.3. HMGB1ΔC Enhances 3D Spheroid Invasion at Lower Concentrations

Similarly to the 2D wound healing assay, to determine the optimal concentrations for 3D spheroid invasion, we first performed a dose–response screening using HMGB1 (100–1000 ng/mL), HMGB1ΔC (100–1000 ng/mL), and TGF-β (1–10 ng/mL) in MDA-MB-231 cells (Figure S2A–C). Based on this screening, the most effective concentrations for promoting invasion were 700 ng/mL HMGB1, 500 ng/mL HMGB1ΔC, and 1 ng/mL TGF-β. Since 500 ng/mL and 700 ng/mL HMGB1ΔC produced comparable invasive responses, 500 ng/mL was selected as the minimal effective concentration to avoid potential saturation or supra-physiological effects. This approach ensured that subsequent functional assays were conducted under conditions that robustly induced invasion while minimizing potential non-specific effects.

**Figure 3:**
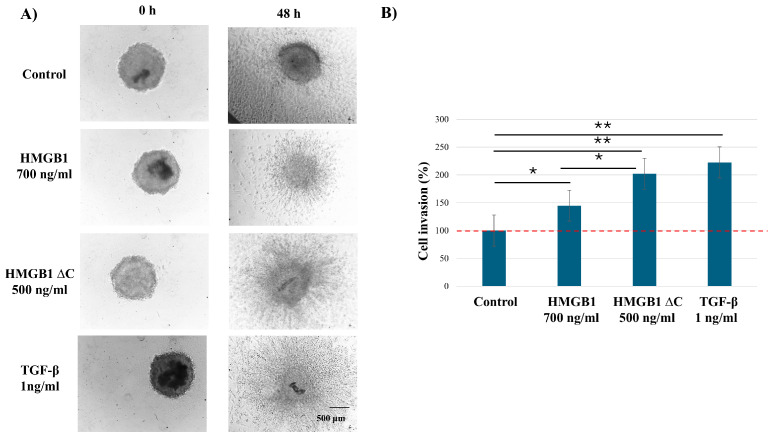
Effect of HMGB1, HMGB1ΔC, and TGF-β on MDA-MB-231 cell invasion. (**A**) Cells were treated with the optimal concentrations of HMGB1, HMGB1ΔC, or TGF-β, and 3D spheroid cell invasion assay was performed. (**B**) Migration was quantified and normalized to the control, which was set as 100% invasion. Data are presented as mean ± SD. Statistical analysis was performed using one-way ANOVA. Statistically significant differences are indicated with * *p* < 0.05 and ** *p* < 0.01.

Our results showed that HMGB1ΔC induced greater spheroid invasion compared to full-length HMGB1 (Figure 3). Furthermore, in the 3D context, removal of the acidic tail was associated with enhanced invasion-related behavior in MDA-MB-231 cells.

### 2.4. HMGB1ΔC Enhances RAGE-Independent Cell Motility, Invasion, and EMT-Associated Traits

Building on existing evidence that the HMGB1 C-terminal tail is critical for the protein binding capabilities of HMGB1 [15], we sought to examine the relative contributions of ligand structure and receptor signaling by silencing RAGE and comparing cellular responses to full-length HMGB1 versus HMGB1ΔC. To do this, we silenced RAGE in MDA-MB-231 cells using esiRNA, achieving approximately 80% knockdown efficiency (Figure S3).

**Figure 4:**
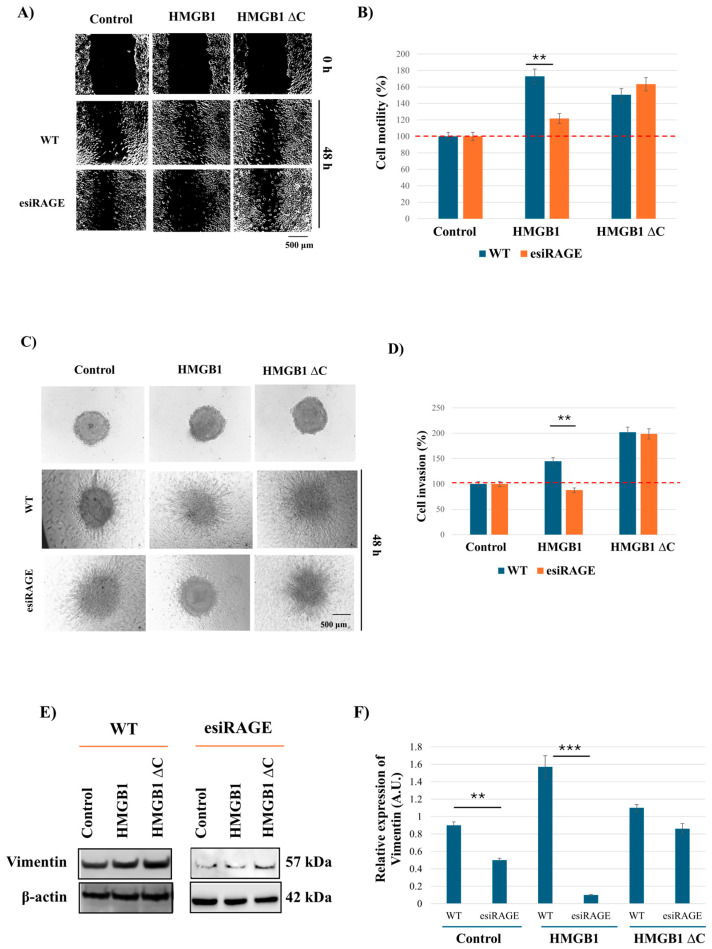
Effect of RAGE silencing on MDA-MB-231 cell motility. (**A**) Representative images of wound healing analysis on wild-type (WT) and RAGE-silenced (esiRAGE) MDA-MB-231 cells at 0 h (before treatment) and 48 h after treatment with 100 ng/mL HMGB1 or 300 ng/mL HMGB1ΔC. (**B**) Quantification analysis of wound closure percentage, normalized to the control (set as 100% wound closure). (**C**) Representative images of invasion assay at 0 h and 48 h in WT and esiRAGE MDA-MB-231 after treatment with 700 ng/mL HMGB1 and 500 ng/mL HMGB1ΔC. (**D**) Quantification analysis for invasion percentage, normalized to the control (set as 100% cell invasion). (**E**) Western blot, showing expression of vimentin in WT and esiRAGE MDA-MB-231 48 h after treatment with 100 ng/mL HMGB1 and 300 ng/mL HMGB1ΔC. (**F**) Quantification analysis of relative vimentin expression. Data shown in arbitrary units (A.U). Data are presented as mean ± SD (n = 3). Statistical analysis was performed using one-way ANOVA. Statistically significant differences are indicated with ** *p* < 0.01 and *** *p* < 0.0001.

In RAGE-silenced cells, treatment with full-length HMGB1 resulted in a marked reduction in cell motility, invasion and vimentin expression compared to control cells, confirming that HMGB1-induced migration and invasion are largely RAGE-dependent. In contrast, HMGB1ΔC maintained its ability to promote motility, invasion and vimentin expression despite RAGE silencing, suggesting that the effects of HMGB1ΔC could occur through a RAGE-independent mechanism (Figure 4). While evaluating additional EMT markers, such as N-cadherin, would further strengthen these observations, the current analysis focused on vimentin as a robust indicator of the mesenchymal phenotype in these cells. These results indicate that the removal of the entire C-terminal tail of HMGB1 may permit the activation of alternative signaling pathways that bypass RAGE in order to enhance EMT-associated features and to drive breast cancer cell motility and invasion.

### 2.5. HMGB1ΔC Enhances NF-κB Activation

Based on prior evidence supporting a central role for NF-κB in HMGB1-mediated EMT and invasion [16], we compared NF-κB activation by assessing the nuclear p-p65 signal in cells treated with HMGB1 and HMGB1ΔC.

**Figure 5:**
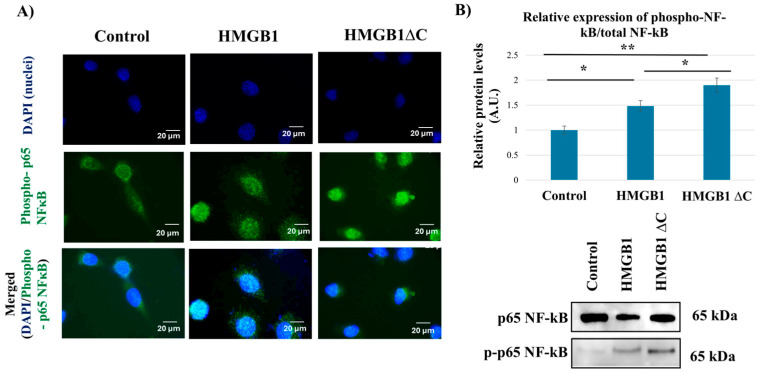
Subcellular translocation and relative expression of phosphorylated p65 NF-κB/total NF-kB after treatment with HMGB1 and HMGB1ΔC. (**A**) Representative images from immunofluorescence analysis on the translocation of phosphorylated p65 NF-κB in MDA-MB-231 cells after treatment with 100 ng/mL HMGB1 and 300 ng/mL HMGB1ΔC for 30 min. Images were captured using an inverted fluorescence microscope AxioVert 200 M (Zeiss) with a 63× oil immersion objective and appropriate green and blue filters. Fluorescence exposure time: 800 ms. Scale bar: 20 μm. (**B**) Representative Western blot analysis of the relative expression levels of phosphorylated p65 NF-κB compared to total p65 in total cell lysates from MDA-MB-231 after a 30 min treatment with 100 ng/mL HMGB1 and 300 ng/mL HMGB1ΔC. Quantification of the relative protein expression levels was performed using densitometric analysis of Western blot signals from three independent experiments (n = 3) using ImageJ software. Phospho-p65 levels were normalized to total p65 from the same lysates. Data are presented as mean ± SD. Statistical analysis was performed using one-way ANOVA. Statistically significant differences are indicated with * *p* < 0.05 and ** *p* < 0.01. Values are presented in arbitrary units (A.U.).

Our immunofluorescence analysis indicated increased nuclear p-p65 signal following HMGB1ΔC treatment compared with HMGB1 (Figure 5A). In support of these findings, Western blot analysis supported this observation, showing increased levels of phosphorylated p65 NF-κB relative to total p65 upon HMGB1ΔC treatment (Figure 5B).

### 2.6. Metformin Inhibits HMGB1-but Not HMGB1ΔC-Induced Motility, Invasion and NF-κB Activation

To evaluate the potential pharmacological inhibition of HMGB1-induced phenotypes, we tested metformin in both 2D monolayer cultures and 3D spheroid models of MDA-MB-231 cells. Metformin has been reported to directly interact with the C-terminal tail of HMGB1 [14], and we previously showed that it can inhibit HMGB1-driven pro-inflammatory and pro-migratory signaling in triple-negative breast cancer [13].

MTT assays revealed that cells cultured as 3D spheroids had reduced sensitivity to metformin compared to those grown in 2D, underscoring the importance of physiologically relevant models for drug testing (Figure S4). Based on these results, five concentrations (IC10, IC20, IC30, IC40, and IC50) were selected for further evaluation.

**Figure 6:**
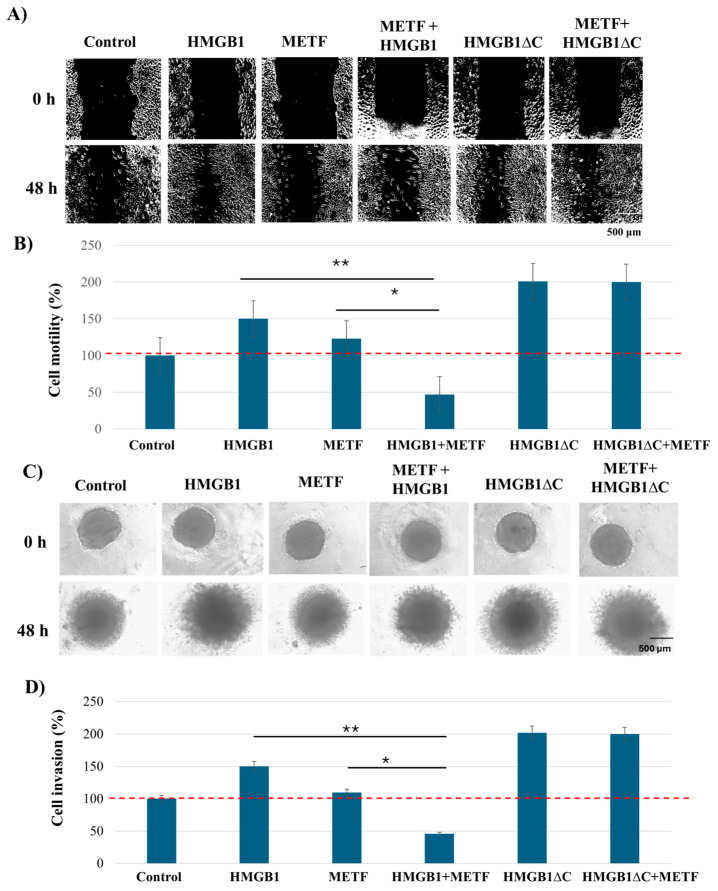

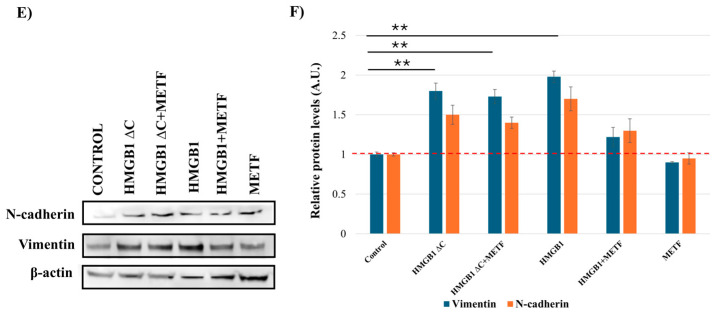
Effect of metformin on HMGB1- and HMGB1ΔC- induced cell motility, invasion and EMT-associated traits in MDA-MB-231 cells. (**A**) Representative images of wound healing assay on MDA-MB-231 cells at 0 h and 48 h after treatment with IC30 metformin alone and in combination with 100 ng/mL HMGB1 and 300 ng/mL HMGB1ΔC are shown; quantification reflects measurements from multiple fields and independent experiments. (**B**) Quantification analysis of wound closure percentage, normalized to the control (set to 100% wound closure). (**C**) Representative images of invasion in collagen matrix at 0 h and 48 h after treatment with IC30 metformin alone and in combination with 700 ng/mL HMGB1 and 500 ng/mL HMGB1ΔC. (**D**) Quantification analysis of invasion percentage, normalized to the control (set as 100% cell invasion). (**E**) Western blot of N-cadherin and Vimentin expression following 48 h treatment with IC30 metformin alone and in combination with 100 ng/mL HMGB1 and 300 ng/mL HMGB1ΔC. (**F**) Quantification of relative expression of N-cadherin and Vimentin, normalized to the control (set as 1). Data shown in arbitrary units (A.U). Data are presented as mean ± SD (n = 3). Statistical analysis was performed using one-way ANOVA. Statistically significant differences are indicated with * *p* < 0.05 and ** *p* < 0.01.

To identify a concentration that effectively inhibited HMGB1-induced motility, invasion, and EMT without causing excessive cytotoxicity, we tested these five inhibitory concentrations in functional assays, including wound healing, 3D spheroid invasion, and analysis of EMT marker (N-cadherin and vimentin) expression. Among the tested concentrations, IC30 consistently produced the most robust inhibitory effects, both alone and in combination with 100 ng/mL HMGB1 and was therefore selected for all subsequent experiments (Figure 6). Notably, none of the concentrations affected baseline motility, invasion, or EMT in HMGB1ΔC, consistent with metformin’s specificity for the C-terminal tail of HMGB1.

### 2.7. Metformin Inhibits HMGB1-Induced Phosphorylation of p65 NF-κB but Has No Effect on HMGB1ΔC

**Figure 7:**
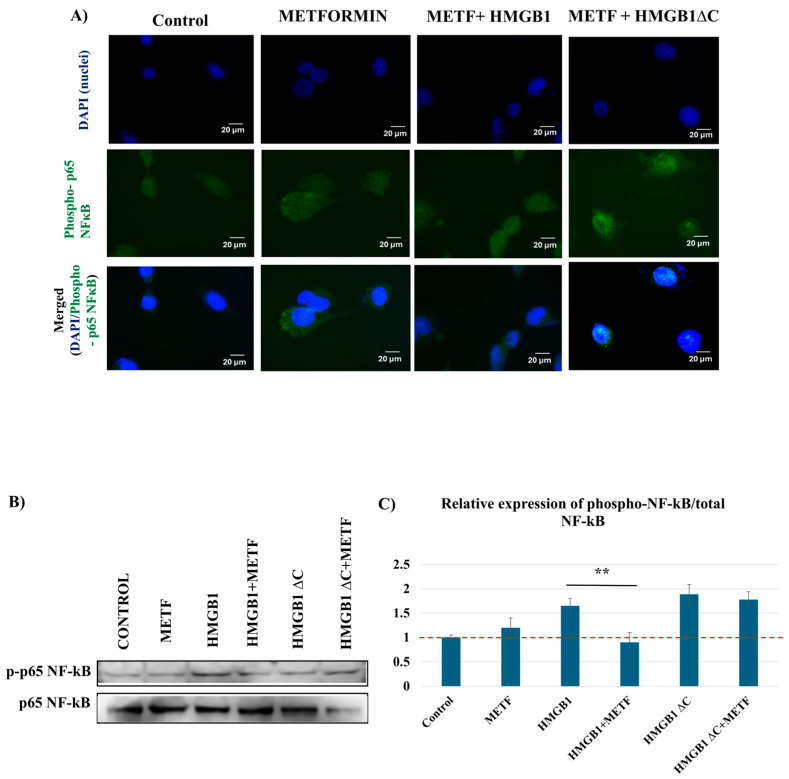
Effect of metformin on subcellular translocation of phosphorylated p65 NF-κB after treatment with IC30 metformin alone and in combination with HMGB1 and HMGB1ΔC. (**A**) Representative images from immunofluorescence analysis showing the effect of IC30 metformin alone and in combination with 100 ng/mL HMGB1 and 300 ng/mL HMGB1ΔC on the translocation of phosphorylated p65 NF-κB after 30 min treatment. Fluorescence exposure time: 870.0 ms. The images were captured using an inverted fluorescence microscope AxioVert 200 M (Zeiss) at 63× magnification with immersion oil, using appropriate filters for the green and blue spectra. Scale bar: 20 μm. (**B**) Representative Western blot analysis of the relative expression levels of phosphorylated p65 NF-κB compared to total p65 in total cell lysates from MDA-MB-231 after a 30 min treatment with IC30 metformin alone and in combination with 100 ng/mL HMGB1 and 300 ng/mL HMGB1ΔC. (**C**) Quantification analysis of phosphorylated p65 NF—κB vs. total p65 NF—κB in total cell lysates. Phospho-p65 levels were normalized to total p65 from the same lysates. Data shown in arbitrary units (A.U). Data are presented as mean ± SD (n = 3). Statistical analysis was performed using one-way ANOVA. Statistically significant differences are indicated with ** *p* < 0.01.

Apart from its inhibitory effects on HMGB1-induced cell motility, invasion, and EMT-associated traits, metformin also reduced HMGB1-induced phosphorylation of p65 NF-κB under the tested conditions, while HMGB1ΔC-associated responses were not substantially affected at the IC30 concentration used in the experiments (Figure 7). These findings are consistent with a potential role of the HMGB1 C-terminal tail in mediating the responsiveness to metformin.

A co-immunoprecipitation experiment was performed as a preliminary assessment of HMGB1 variant association with RAGE; the data are presented in Supplementary Figure S6. MDA-MB-231 cells were treated for 48 h with recombinant full-length HMGB1, a combination of full-length HMGB1 and metformin, or a truncated HMGB1 variant lacking the C-terminal tail (HMGB1ΔC). Control cells were cultured in growth medium without recombinant proteins. Protein complexes were immunoprecipitated using an anti-RAGE antibody and analyzed by SDS-PAGE followed by immunoblotting with an anti-HMGB1 antibody. Reprobing of the membrane with an anti-RAGE antibody confirmed the presence of RAGE in all immunoprecipitated fractions. A signal corresponding to HMGB1 was detected in samples treated with full-length HMGB1, whereas the signal appeared reduced in cells co-treated with HMGB1 and metformin and a weaker signal was observed in HMGB1ΔC-treated samples (Figure S6).

Given the absence of key Co-IP controls, these data are considered preliminary and do not allow definitive conclusions regarding binding specificity or interaction strength. Therefore, the Co-IP experiment is presented only as a supplementary biochemical observation.

## 3. Discussion

In this study, we investigated the effects of HMGB1 lacking the C-terminal acidic tail (HMGB1ΔC) on EMT-associated traits, cell motility, and invasion in the triple-negative breast cancer cell line MDA-MB-231 and assessed the ability of metformin to modulate these phenotypes. HMGB1ΔC treatment was associated with increased motility, invasion, and elevated expression of mesenchymal markers such as N-cadherin and vimentin. These effects were accompanied by enhanced phosphorylation of NF-κB p65, consistent with its role in EMT-associated transcriptional programs and matrix remodeling [17,18,19].

Functional experiments demonstrated that RAGE silencing attenuated the pro-migratory and pro-invasive effects of full-length HMGB1, whereas HMGB1ΔC-induced phenotypes persisted under RAGE downregulation. These findings suggest that, in this cellular context, HMGB1ΔC-driven signaling is not strictly dependent on RAGE expression.

The co-immunoprecipitation experiment (Supplementary Figure S6) provided limited preliminary information regarding the presence of HMGB1 variants in the RAGE-immunoprecipitated fraction under the tested conditions. Due to the absence of key experimental controls, these data do not allow definitive conclusions regarding receptor binding or interaction specificity. Additional experiments, including appropriate controls and orthogonal approaches, will be required to fully characterize HMGB1ΔC receptor interactions.

Therefore, these results are interpreted as preliminary biochemical observations and considered alongside the functional RAGE-silencing data. Together, the findings raise the possibility that truncation of the C-terminal acidic tail may alter receptor interaction dynamics and facilitate engagement of alternative signaling pathways, potentially involving other pattern recognition receptors such as TLR2 or TLR4 [20].

The C-terminal acidic tail of HMGB1 has been proposed to regulate the accessibility and interaction capacity of the HMG boxes through intramolecular interactions that can partially shield functional domains. In this context, removal of the C-terminal tail may increase the availability of HMGB1 for receptor engagement and alter its signaling properties. Consistent with previous reports [21] demonstrating enhanced biological activity of truncated HMGB1 variants in cancer cells, our findings suggest that HMGB1ΔC may promote a more aggressive cellular phenotype by facilitating alternative receptor interactions and downstream signaling pathways.

Using 3D spheroid models, we observed that HMGB1ΔC induced a stronger invasive response compared to full-length HMGB1 under the tested conditions, despite differences in the absolute ligand concentrations required for each protein. This indicates that removal of the C-terminal tail is associated with enhanced invasion-related behavior in MDA-MB-231 cells and aligns with previous observations of altered signaling properties of HMGB1ΔC in inflammatory models [22].

The mechanism underlying the generation of truncated HMGB1 forms in tumor contexts remains unclear. However, it is plausible that HMGB1 may undergo proteolytic processing under conditions associated with the tumor microenvironment, including chronic inflammation, oxidative stress, or increased protease activity. Such processing could generate truncated variants with altered structural and functional properties. In particular, removal of the C-terminal acidic tail may disrupt intramolecular regulation and shift HMGB1 toward a more active conformation, thereby enhancing its ability to modulate cancer-associated phenotypes. Together, these findings suggest that structural modification of HMGB1 may not only alter receptor engagement but also influence its susceptibility to pharmacological intervention.

Metformin effectively inhibited full-length HMGB1-induced motility, invasion, and NF-κB phosphorylation, in agreement with prior reports of interaction with the C-terminal region of HMGB1 [13,14]. In contrast, HMGB1ΔC-mediated phenotypes were largely unaffected at the IC_30_ concentration tested. These findings suggest that truncation of the acidic tail may contribute to reduced pharmacological responsiveness.

A limitation of the present study is that all experiments were conducted in a single cell line (MDA-MB-231); therefore, further validation in additional breast cancer models will be required. In conclusion, our results suggest that removal of the C-terminal acidic tail of HMGB1 enhances EMT-associated traits, motility, and invasion in triple-negative breast cancer cells, potentially through RAGE-independent mechanisms and altered receptor interaction dynamics. These findings provide new insight into the regulatory role of HMGB1 structural domains in cancer-associated phenotypes and highlight the potential relevance of HMGB1 truncation as a previously underappreciated mechanism contributing to tumor progression and therapeutic resistance.

## 4. Materials and Methods

### 4.1. Cell Culture

The human breast cancer cell line MDA-MB-231 (TNBC cell line) was acquired from the American Type Culture Collection (ATCC, Manassas, VA, USA). MDA-MB-231 cells were cultivated in DMEM, high glucose (Cat# 11965092, Gibco™, Thermo Fisher Scientific, Waltham, MA, USA) media supplemented with 10% FBS (Fetal Bovine Serum), certified (Cat# 16000044, Gibco™, Thermo Fisher Scientific, Waltham, MA, USA) and 1% antibiotics (Antibiotic Antimycotic Solution) (100×), Stabilized (Cat# A5955, Sigma-Aldrich, St. Louis, MO, USA). The cells were grown at a controlled temperature of 37 °C with 5% CO_2_. The cells used for the experiments were between 2 and 8 passages. DAPI staining did not show any sign of Mycoplasma contamination.

### 4.2. Isolation and Purification of Recombinant HMGB1 and HMGB1ΔC

HMGB1 (214 aa) and HMGB1ΔC (C-terminally truncated variant (1–184 aa)) were cloned in pET28a+ plasmid (pET-28a(+) DNA) (Cat# 69864-3, Novagen^®^, MilliporeSigma, Burlington, MA, USA) was expressed in modified *Escherichia coli* BL21 Poly Lys S as previously described [23]. The presence of His-tag on the recombinant proteins allowed for purification via His-tag resin (HIS-Select^®^ Nickel Affinity Gel) (Cat# P6611, Millipore, MilliporeSigma, Burlington, MA, USA) according to the manufacturer’s protocol (Figure S5). To ensure the removal of endotoxins, an on-column wash was performed while the His-tagged protein was bound to the Ni-NTA resin. The column was washed with equilibration buffer supplemented with 1% Triton X-114 (Sigma-Aldrich).

### 4.3. Wound Healing Assay

Cell migration was assessed using the wound healing assay, as previously described [24]. Briefly, cells were seeded in 6-well plates and cultured to confluence. A linear scratch was introduced using a sterile pipette tip, and the medium was replaced with low-serum medium (0.1% FBS) to minimize proliferation. Recombinant HMGB1 and HMGB1ΔC were applied at concentrations of 100, 300, 500, 700, and 1000 ng/mL. Wound closure was imaged at 0, 24, and 48 h using an inverted phase-contrast microscope at 10× objective (Zeiss AxioVert 200 M; Zeiss, Oberkochen, Germany). Wound widths were measured at three fixed points per well, and the percentage of closure was calculated. Data were analyzed using ImageJ software (bundled with 64-bit Java 8, National Institutes of Health, Bethesda, MD, USA; https://imagej.nih.gov/ij/, version 1.54p, accessed on 17 February 2025) and statistical analysis was performed with Microsoft Excel, applying one-way ANOVA with significance set at *p* < 0.05.

### 4.4. Three-Dimensional Tumor Spheroid Invasion Assay

Three-dimensional spheroids were prepared according to a standard protocol with methylcellulose [25]. Invasive potential was evaluated using a 3D tumor spheroid invasion model in type I collagen, as previously described [26]. Collagen was neutralized on ice to 2.5 mg/mL (pH 7.2–7.4) using 1N NaOH, 10× PBS, and sterile water. Thirty microliters of collagen were added to each well of a 96-well plate, and fully formed spheroids (72 h culture in methylcellulose-containing medium) were positioned on top. A second 30 µL collagen layer was added to create a 3D sandwich, and plates were incubated at 37 °C, 5% CO_2_ for 1 h to allow gelation. Following gelation, the same concentrations of HMGB1 and HMGB1ΔC were tested in serum-reduced media, similarly to the set-up for wound healing assay. Invasion (formation of pseudopodia) was monitored at 0, 24, and 48 h using inverted phase-contrast microscopy at 10× objective (Zeiss AxioVert 200 M; Zeiss, Oberkochen, Germany). Quantitative analysis of invasion was performed using ImageJ (Java 8.0), measuring both the total invasion area and the radial outgrowth beyond the initial spheroid boundary. Experiments were performed in three independent biological replicates, with data expressed as mean ± SD. Statistical significance was determined using one-way ANOVA (*p* < 0.05).

### 4.5. Western Blot

Cell lysates were prepared and analyzed by Western blotting as previously described [27], with minor modifications. Cells were lysed in ice-cold RIPA buffer (50 mM Tris-HCl pH 7.5, 150 mM NaCl, 1% NP-40, 0.5% sodium deoxycholate, 0.1% SDS) supplemented with protease (cOmplete™, EDTA-free; Roche, Cat# 4693132001) and phosphatase inhibitors (Halt™ Phosphatase Inhibitor Cocktail, Thermo Scientific™, Cat# 78440). Lysates were cleared by centrifugation at 7000× *g* for 15 min at 4 °C, and protein concentrations were determined using a Bradford assay.

Equal amounts of protein (20 µg) were resolved on 10% SDS–PAGE gels. In some cases, to facilitate simultaneous detection of different target proteins under identical conditions, 20 µg from the same lysates were loaded onto parallel gels. Following electrophoresis, proteins were transferred to nitrocellulose membranes (Immobilon-P, Millipore) or PVDF membranes (activated for 2 min in 100% methanol) for phosphorylated p65-NF-κB detection, using a wet transfer system (Bio-Rad, Hercules, CA, USA). Membranes were blocked for 1 h at room temperature in 5% BSA in Tris-buffered saline with 0.1% Tween-20 (TBST).

Primary antibodies were incubated overnight at 4 °C in 5% BSA at the manufacturer’s recommended dilutions. The used antibodies in this study included anti-N-cadherin (D4R1H) XP^®^ Rabbit mAb (Cat#13116S; Cell Signaling Technology, Danvers, MA, USA) at dilution 1:1000, anti-Vimentin (D21H3) XP^®^ Rabbit mAb (Cat# 5741S; Cell Signaling Technology, Danvers, MA, USA) at dilution 1:1000, anti-p65-NF-κB (D14E12) XP^®^ Rabbit mAb (Cat# 8242; Cell Signaling Technology, Danvers, MA, USA) at dilution 1:500, anti-phospho-p65-NF-κB (Ser536) (93H1) Rabbit mAb (Cat# 3033S; Cell Signaling Technology, Danvers, MA, USA) at dilution 1:500, anti-RAGE (Cat # ab216329; Abcam, Cambridge, MA, USA) at dilution 1:1000, and anti-β-actin (Cat # A2228; Sigma-Aldrich, St. Louis, MO, USA) at dilution 1:2000. Following overnight incubation, the membranes were washed three times with TBST for 10 min each, after which they were incubated with HRP-conjugated secondary antibodies at dilution 1:10,000 for 1 h at room temperature in dark conditions. The HRP-conjugated antibodies in this study were HRP-conjugated goat anti-mouse IgG (H+L) secondary antibody (Cat# 31430; Invitrogen™, Thermo Fisher Scientific, Waltham, MA, USA) and HRP-conjugated goat anti-rabbit IgG (H+L) secondary antibody (Cat# 31460; Invitrogen™, Thermo Fisher Scientific, Waltham, MA, USA). The membranes were washed again with TBST three times for 10 min each, incubated with SuperSignal™ West Pico PLUS (Cat #34580; Thermo Scientific™, Waltham, MA, USA) for 5 min and the immunoreactive bands were visualized using C-DiGit^®^ Blot Scanner (LI-COR Biotech, LLC; Lincoln, NE, USA). Band intensities were quantified by densitometry using ImageJ (Java 8.0), with target protein signals normalized to loading controls (β-actin). Protein concentrations were determined using Bradford assay, and equal amounts of total protein were loaded per lane. For NF-κB signaling analyses, phospho-p65 levels were quantified relative to total p65 from the same lysates and membranes providing internal normalization of pathway activation.

### 4.6. Immunofluorescence

Immunofluorescence was performed according to a standard protocol [28], with minor modifications. Approximately 30,000 cells were seeded onto sterile glass coverslips and cultured under standard conditions until ~70% confluence. For staining, cells were fixed with 4% paraformaldehyde in PBS for 20 min at room temperature and blocked with blocking buffer (10% fetal calf serum, 1% BSA, and 0.1% Triton X-100 in PBS) for 1 h. Following this, the coverslips were incubated with the primary antibodies at 4 °C overnight. The primary antibody used in this study is anti-phospho-p65-NF-κB (Ser536) (93H1) Rabbit mAb (Cat# 3033S; Cell Signaling Technology, Danvers, MA, USA) at dilution 1:200. After three washes (10 min each) in PBS, cells were incubated with Alexa Fluor 488-conjugated polyclonal secondary antibody (Cat# A-11094; Invitrogen™, Thermo Fisher Scientific, Waltham, MA, USA) at dilution 1:2000 for 1 h at 37 °C. After two washes in PBS, the coverslips were rinsed in distilled water and briefly dipped in 100% ethanol. After a quick dry, the coverslips were mounted on a slide with Fluoromount-G™ Mounting Medium (Cat# 00-4958-02, Invitrogen™, Thermo Fisher Scientific, Waltham, MA, USA) containing 400 ng/mL DAPI. Images were taken using an epifluorescence microscope Zeiss AxioVert 200 M (Zeiss, Oberkochen, Germany) using a 63× objective with oil immersion.

### 4.7. RAGE Silencing Using RNA Interference

Endoribonuclease-prepared siRNAs (esiRNAs) specifically targeting the coding regions of RAGE were synthesized using an endoribonuclease preparation method, as previously described [29,30]. Primers for targeting the specific regions of RAGE were selected from the Riddle database [29]. The primer sequences used were as follows: forward primer 5′-TCACTATAGGGAGAGGATCCAGGATGAGGGGATTT and reverse primer 5′-TCACTATAGGGAGACGCTACTGCTCCACCTTCTGG. For the RNA interference experiment, each well of a 24-well plate with a transfection volume of 500 µL contained 30 pmol of esiRNA and 2 µL of Lipofectamine 2000 (Thermo Fisher Scientific, Waltham, MA, USA). The knockdown efficiency of RAGE was assessed using Western blotting analysis.

### 4.8. MTT Analysis

MTT assays were performed in both 2D monolayer and 3D spheroid cultures of MDA-MB-231 cells, according to a standard protocol [31], with modifications regarding the 3D spheroids. For the 2D assay, cells were seeded in 96-well plates at 3000 cells per well (30,000 cells/mL; 100 µL per well) and allowed to adhere for 24 h at 37 °C in a humidified atmosphere with 5% CO_2_ before treatment with metformin (0.625–80 mM) for 72 h. For the 3D assay, cells were suspended in medium containing 2.5 mg/mL methylcellulose at 30,000 cells/mL and seeded into ultra-low-attachment, round-bottom 96-well plates (100 µL per well) to allow spheroid formation; mature spheroids formed within 72 h and were treated with the same concentrations of metformin for 96 h. At the end of treatment, cell viability was assessed by adding serum-free medium containing MTT (0.5 mg/mL) to each well (100 µL). Incubation was carried out for 3 h for monolayers and 6 h for spheroids to account for differences in structure. The MTT solution was then removed, and formazan crystals were solubilized with 100 µL 100% DMSO; in the case of spheroids, mechanical disruption by pipetting was performed to ensure complete dissolution. Plates were incubated on a shaker (300 rpm, 30 min, room temperature, protected from light), and absorbance was measured at 570 nm with reference at 630 nm using a Varioskan™ multimode plate reader (Thermo Scientific™, Waltham, MA, USA). Blank values (DMSO without cells) were subtracted, and data were normalized to untreated controls set at 100% viability. Results represent mean ± standard deviation from at least three independent experiments, each performed in technical triplicate.

### 4.9. Co-Immunoprecipitation Using Protein A/G Magnetic Beads

Co-immunoprecipitation (Co-IP) was performed to assess the interaction between HMGB1 and RAGE under conditions with or without metformin, following a protocol of the manufacturer Pierce Biotechnology, with minor modifications. MDA-MB-231 cells were cultured in T75 flasks under standard conditions (37 °C, 5% CO_2_) and treated with 100 ng/mL recombinant HMGB1 and 300 ng/mL HMGB1ΔC for 48 h; in parallel, one set of cells was co-treated with HMGB1 and metformin at the IC_30_ concentration. After treatment, cells were washed twice with 1× PBS, harvested by trypsinization, lysed in ice-cold RIPA buffer for 40 min, and sonicated with three 5 s pulses. Lysates were clarified by centrifugation at 14,000× *g* for 10 min at 4 °C, and the supernatants containing soluble proteins were collected. For immunoprecipitation, 25 µL of Pierce Protein A/G magnetic beads (Cat# 88802; Thermo Scientific™, Waltham, MA, USA) were prewashed in TBS containing 0.05% Tween-20, and incubated with lysates pre-mixed with anti-RAGE antibody (2 µg) for 1 h at room temperature with rotation. Beads were captured on a magnetic rack, and unbound fractions were retained for analysis. Beads were washed three times with 500 µL wash buffer (50 mM Tris-HCl, 0.05% Tween-20, 0.5 M NaCl) and once with distilled water. Bound proteins were eluted with 100 µL Laemmli buffer (10% SDS, 20% glycerol, 125 mM Tris-HCl, 1% β-mercaptoethanol, 0.02% bromophenol blue, pH 6.8) by heating at 95 °C for 5 min. Eluates (30 µL per lane) were resolved on 10% SDS-PAGE and analyzed by Western blotting. Membranes were first probed with anti-RAGE (Cat # ab216329; Abcam, Cambridge, MA, USA) at dilution 1:1000 to confirm successful co-immunoprecipitation and subsequently with anti-HMGB1 (Cat # ab18256; Abcam, Cambridge, MA, USA) at dilution 1:1000 to detect HMGB1 in the immunoprecipitated fraction.

### 4.10. Statistical Analysis

Data is presented as mean ± SD of three independent experiments. One-way ANOVA, performed via Graph Pad Prism8 (San Diego, CA, USA) was used to compare the means between groups. *p*-values < 0.05 were indicative of results with statistically significant difference.

## Data Availability

The original contributions presented in this study are included in the article and Supplementary Materials. Further inquiries can be directed to the corresponding authors.

## Supplementary Materials

The following supporting information can be downloaded at: https://www.mdpi.com/article/10.3390/ijms27073146/s1.
