# Supplementary material for: The Role of the HMGB1 C-Terminal Domain in Epithelial–Mesenchymal Transition and Invasion in 2D and 3D MDA-MB-231 Breast Cancer Models
Source: Int J Mol Sci. 2026 Mar 30;27(7):3146. doi: 10.3390/ijms27073146 (PMC13072789; doi:10.3390/ijms27073146)
Supplement: Supplementary file 1 [file ijms-27-03146-s001.zip › Legends to Supplementary Figures-2026.pdf]

Text to Supplementary Figures

S.1 A - C: Comparative analysis of cell movement in a 2D cell model upon treatment with HMGB1full, HMGB1deltaC and TGFbeta

S.2 A – C: Comparative analysis of cell movement in a 3D cell model upon treatment with HMGB1full, HMGB1deltaC and TGFbeta

S.3 - Silenced RAGE with siRNA in MDA-MB-231 cancer cell line

S.4. - MTT assay to determine the IC50 of metformin on MDA-MB-231 cancer cells

S.5. - SDS PAGE of purified recombinant HMGB1full length and HMGB1-delta C

S.6.- Co-immunoprecipitation analysis of recombinant HMGB1 variants precipitated with RAGE in MDA-MB-231 cells and the effect of metformin. Line 1 – untreated cells; Line 2 – cells co-treated with full-length HMGB1 and metformin; Line 3 – cells treated with full-length HMGB1 alone; Line 4 – cells treated with truncated HMGB1ΔC.
