# Supplementary figures and images for: The Role of the HMGB1 C-Terminal Domain in Epithelial–Mesenchymal Transition and Invasion in 2D and 3D MDA-MB-231 Breast Cancer Models
Source: Int J Mol Sci. 2026 Mar 30;27(7):3146. doi: 10.3390/ijms27073146 (PMC13072789; doi:10.3390/ijms27073146)

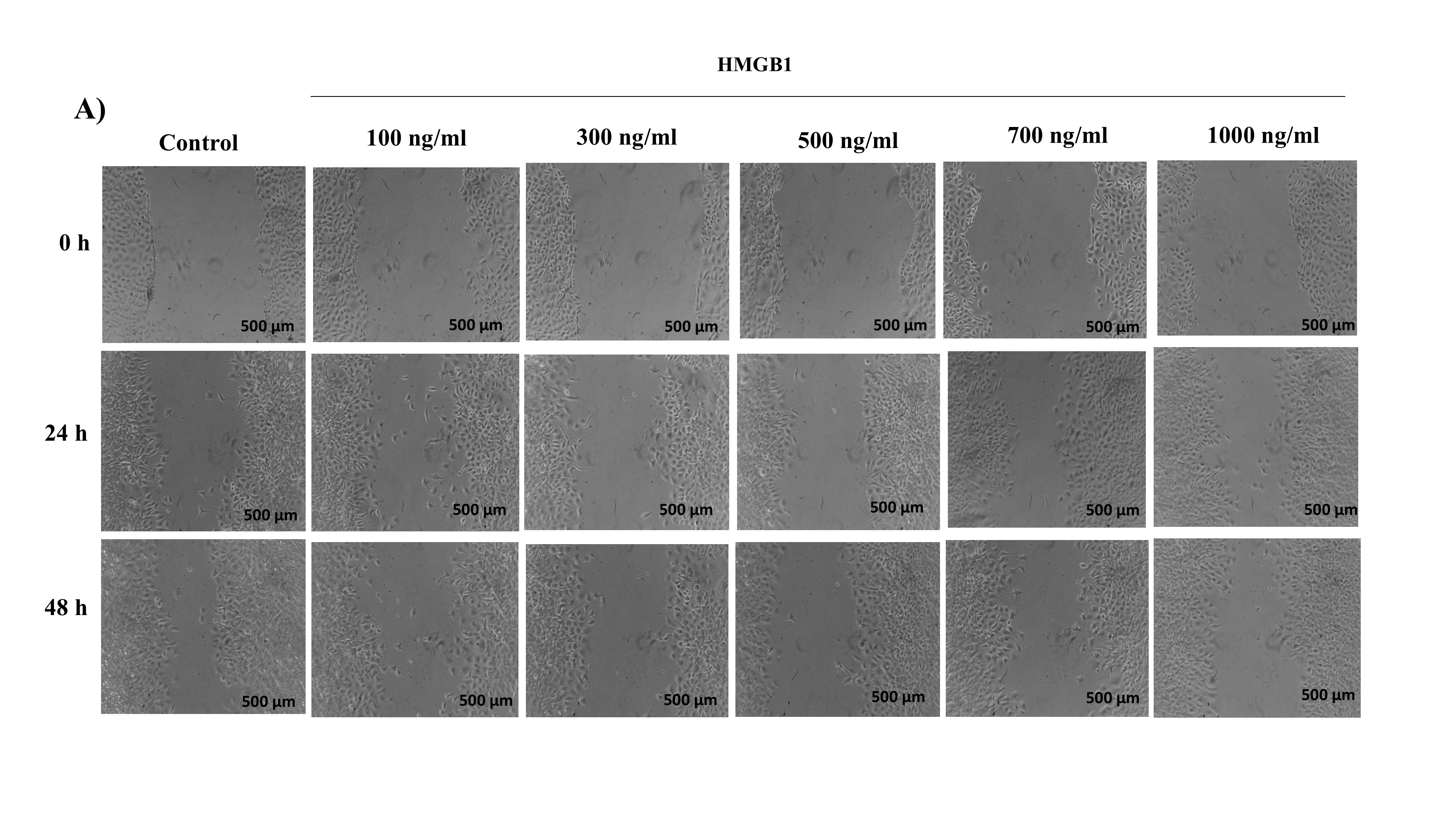

Supplement: Supplementary file 1 [file ijms-27-03146-s001.zip › Figure S1 A.PNG]

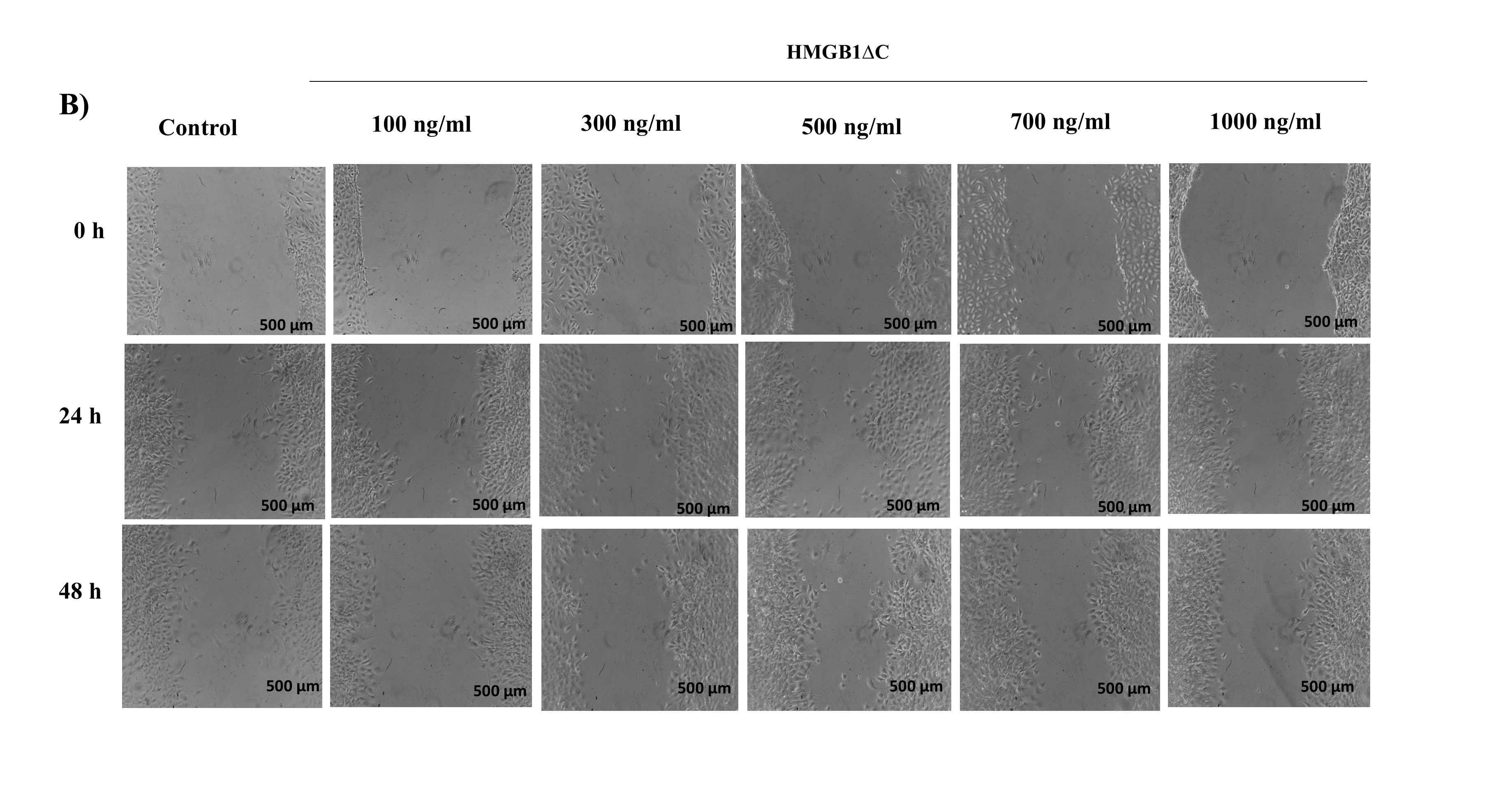

Supplement: Supplementary file 1 [file ijms-27-03146-s001.zip › Figure S1 B.PNG]

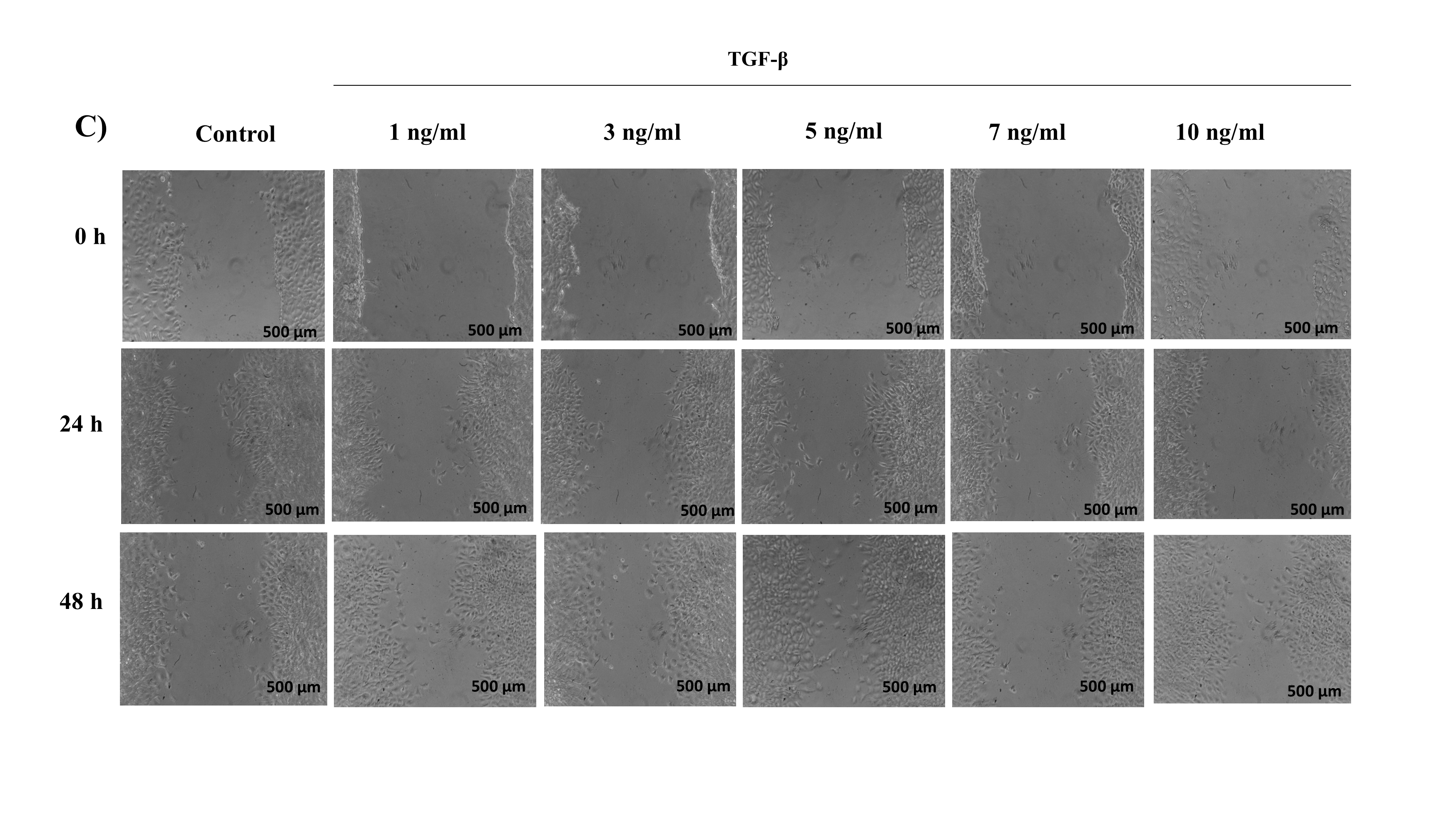

Supplement: Supplementary file 1 [file ijms-27-03146-s001.zip › Figure S1 C.PNG]

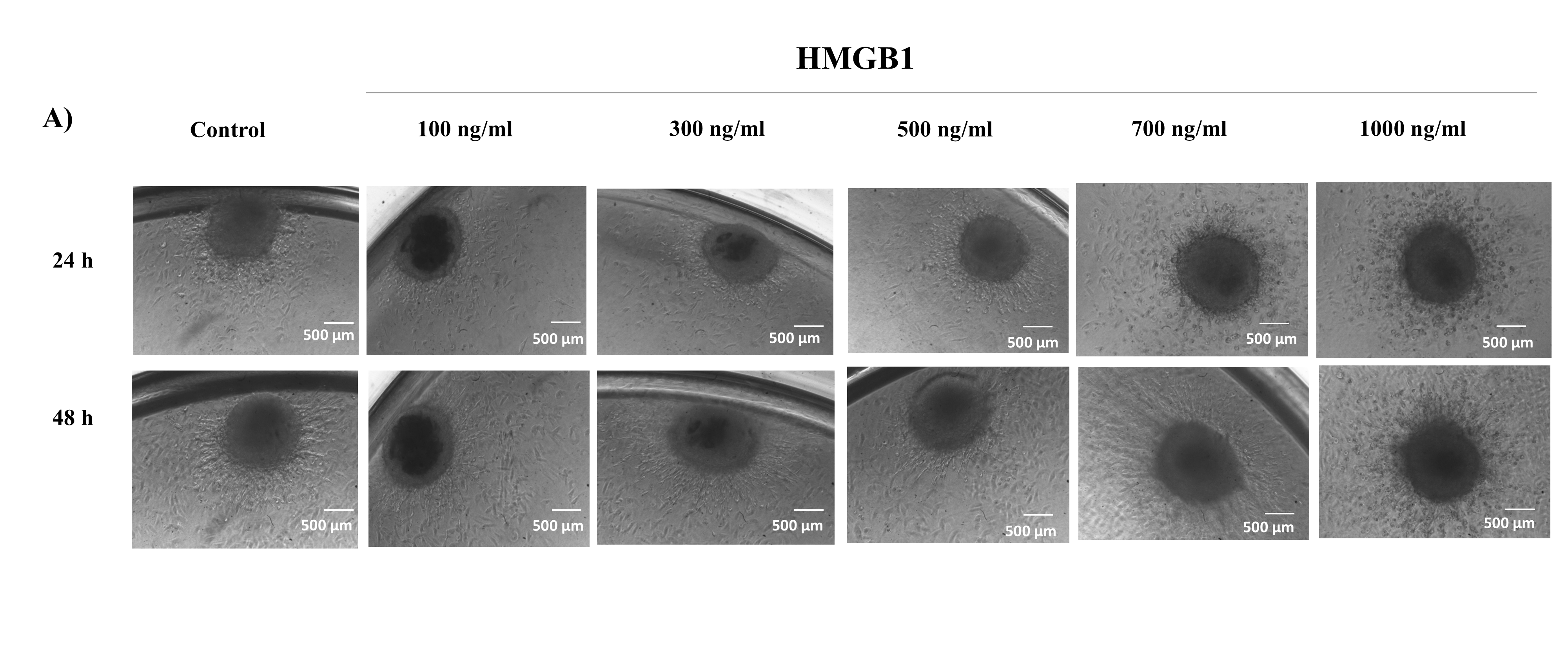

Supplement: Supplementary file 1 [file ijms-27-03146-s001.zip › Figure S2 A.PNG]

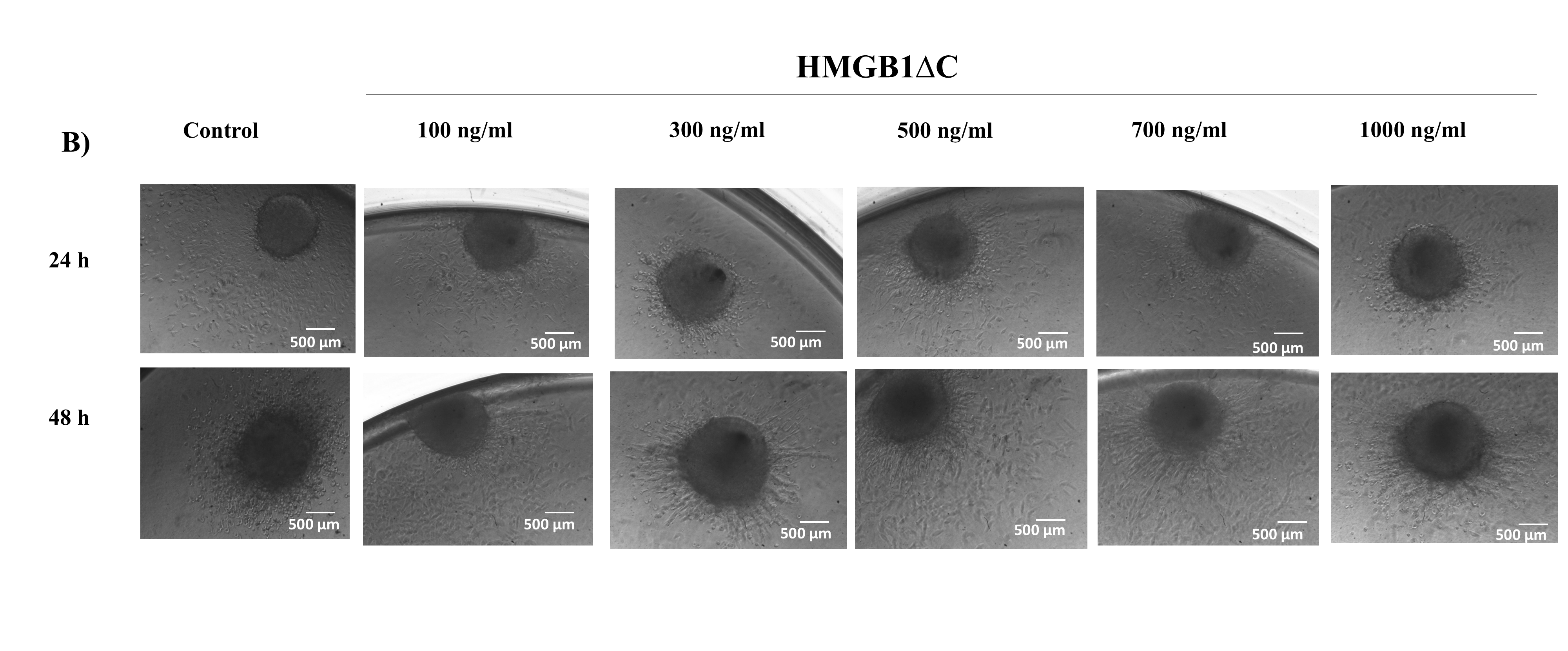

Supplement: Supplementary file 1 [file ijms-27-03146-s001.zip › Figure S2 B.PNG]

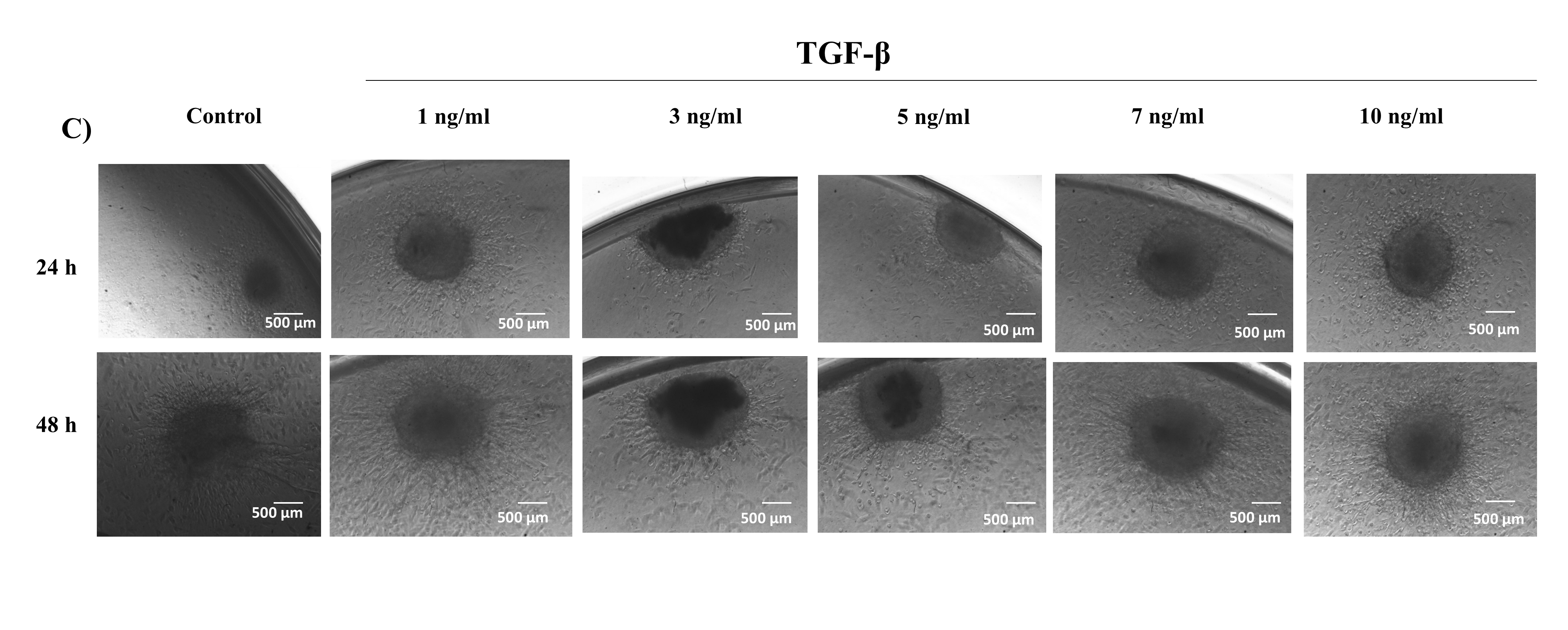

Supplement: Supplementary file 1 [file ijms-27-03146-s001.zip › Figure S2 C.PNG]

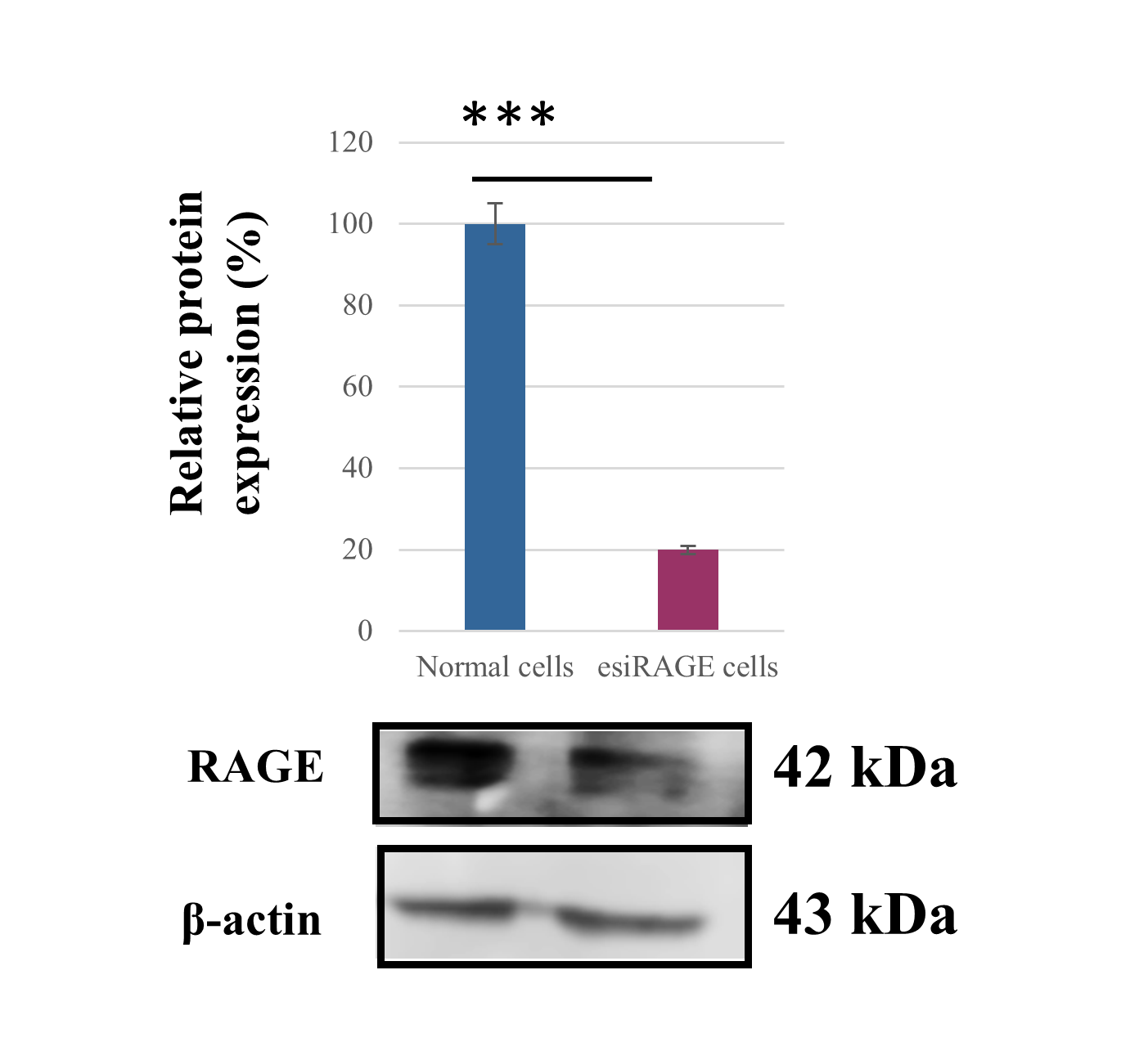

Supplement: Supplementary file 1 [file ijms-27-03146-s001.zip › Figure S3.PNG]

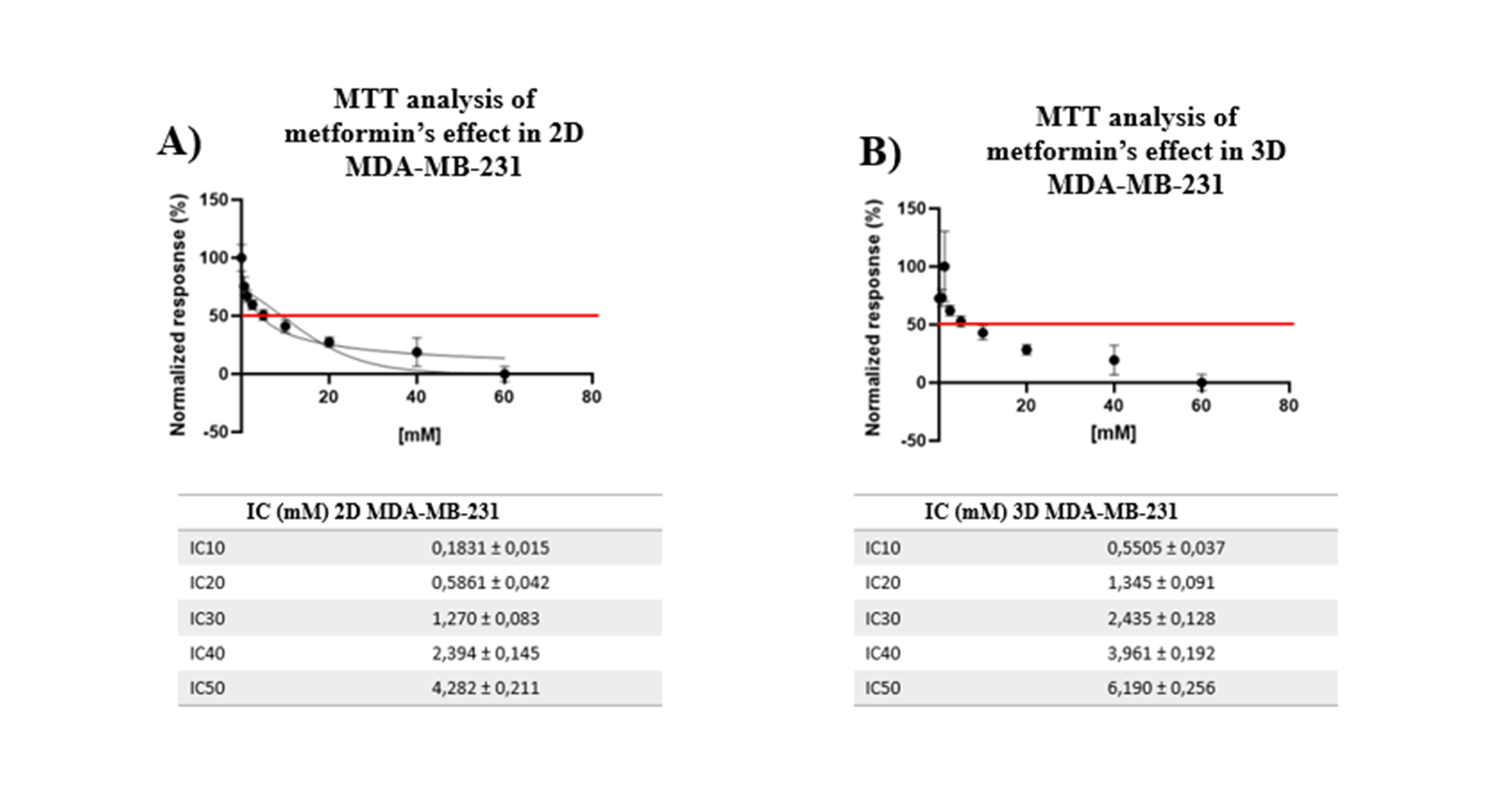

Supplement: Supplementary file 1 [file ijms-27-03146-s001.zip › Figure S4.PNG]

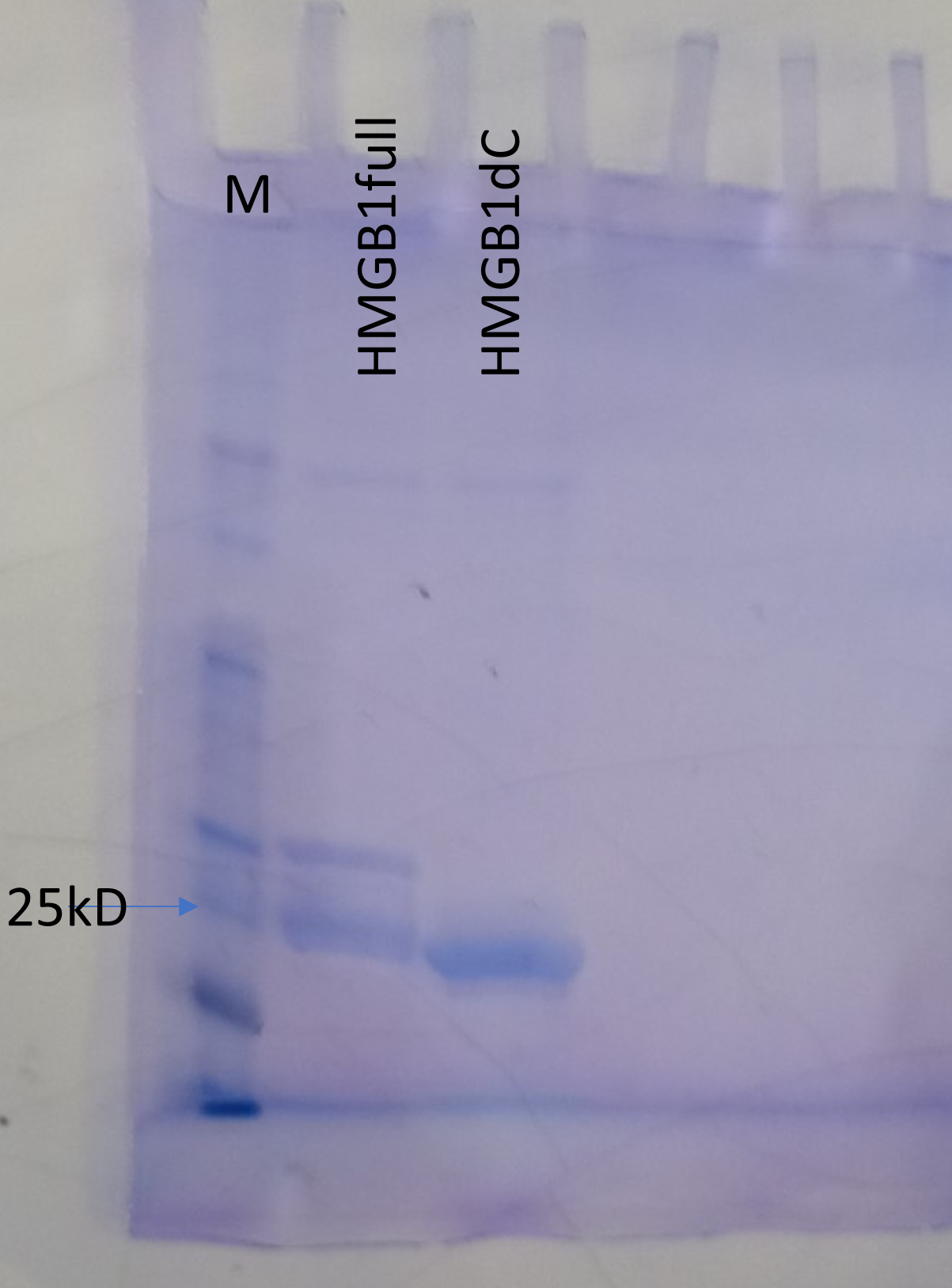

Supplement: Supplementary file 1 [file ijms-27-03146-s001.zip › Figure S5.PNG]

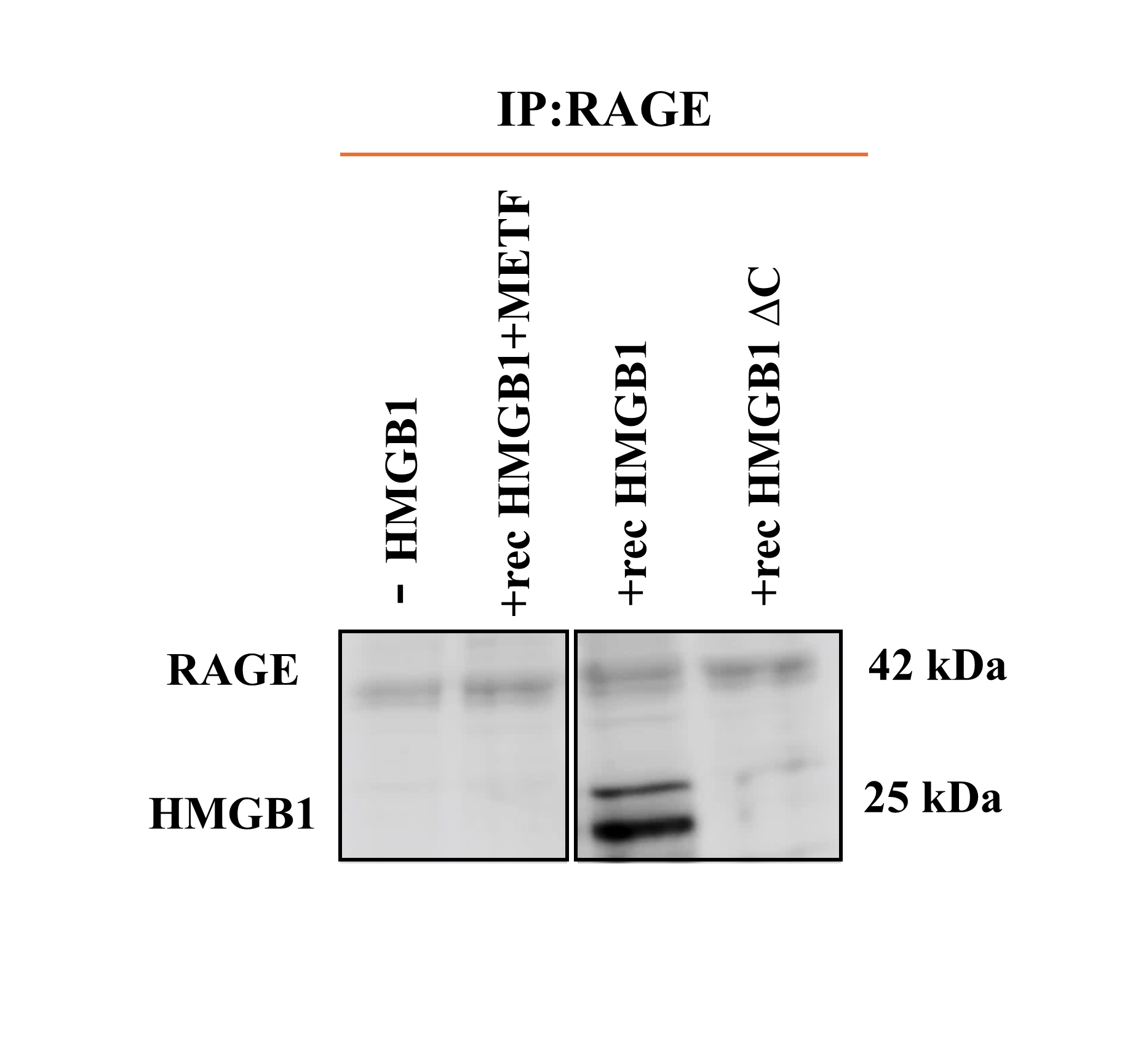

Supplement: Supplementary file 1 [file ijms-27-03146-s001.zip › Figure S6.PNG]
